# Fabrication of Yttrium Oxide Hollow Films for Efficient Passive Radiative Cooling

**DOI:** 10.3390/ma16237373

**Published:** 2023-11-27

**Authors:** Heegyeom Jeon, Sohyeon Sung, Jeehoon Yu, Hyun Kim, Yong Seok Kim, Youngjae Yoo

**Affiliations:** 1 Department of Advanced Materials Engineering, Chung-Ang University, Anseong 17546, Republic of Korea; avel004@cau.ac.kr (H.J.); yujeehoon@cau.ac.kr (J.Y.); 2 Advanced Materials Division, Korea Research Institute of Chemical Technology (KRICT), Daejeon 34114, Republic of Korea; gus6319@krict.re.kr (S.S.); hyunkim@krict.re.kr (H.K.); yongskim@krict.re.kr (Y.S.K.)

**Keywords:** passive radiative cooling, polydimethylsiloxane, yttrium oxide, solar reflectivity, long-wavelength infrared emissivity

## Abstract

In recent years, many parts of the world have researched the transition to renewable energy, reducing energy consumption and moving away from fossil fuels. Among the studies to reduce energy consumption, passive radiative cooling can reduce the energy used for building cooling, and to improve this, the optical properties of atmospheric window emissivity and solar reflectance must be increased. In this study, hollow yttrium oxide (H-Y_2_O_3_) was fabricated using melamine formaldehyde (MF) as a sacrificial template to improve the optical properties of passive radiative cooling. We then used finite-difference time-domain (FDTD) simulations to predict the optical properties of the fabricated particles. This study compares the properties of MF@Y(OH)CO_3_ and H-Y_2_O_3_ particles derived from the same process. H-Y_2_O_3_ was found to have a solar reflectance of 70.73% and an atmospheric window emissivity of 86.24%, and the field tests revealed that the temperature of MF@Y(OH)CO_3_ was relatively low during the daytime. At night, the temperature of the H-Y_2_O_3_ film was found to be 2.6 °C lower than the ambient temperature of 28.8 °C. The optical properties and actual cooling capabilities of the particles at each stage of manufacturing the hollow particles were confirmed and the cooling capabilities were quantified.

## 1. Introduction

When indoor temperatures are unsuitable for human habitation, such as in summer or winter, energy is generated from fossil fuels to control the temperature and maintain it above or below the ambient temperature. Most of the consumed energy is in the form of electricity, and nowadays most of the electrical energy is produced using fossil fuels. The heat generated during this process and the greenhouse gases produced by combustion contribute to global warming [1]. To address this, global agreements are being forged to reduce greenhouse gas emissions, and researchers are studying how to decrease energy consumption. Such research has led to the emergence of passive radiative cooling (PRC), a concept for cooling without energy consumption. PRC can be used to reduce the temperature of buildings or vehicles used for human habitation [2], as well as clothing [3], and unlike other temperature-controlled cooling methods, it refers to a phenomenon that involves cooling without an energy source [4,5]. The principle of PRC encompasses the release of heat energy into space via electromagnetic radiation, where energy transfer occurs without a heat transfer source. At this time, the heat energy must be transferred into space without being absorbed into the Earth’s atmosphere to radiate energy into space. The heat energy coming from the outside must be less than the heat energy emitted via radiation. To satisfy this requirement, energy must radiate in a wavelength range that cannot be absorbed by the atmosphere, and solar energy, which is the largest source of heat energy, must be blocked. Therefore, in previous studies, organic and inorganic particles were added to a polymer matrix to produce a composite, and the cooling performance of this coating was confirmed. Studies have also been conducted on the structure of materials used for PRC. To achieve PRC, studies using both organic and inorganic materials have been conducted to fabricate multilayers [6,7]: an organic layer with Y_2_O_3_ added on a SiC substrate [8], an organic layer with cellulose and silica added on an Ag reflection layer [9], and a structure in which a Si_3_N_4_ layer and MgF_2_ are stacked multiple times on an Ag reflection layer [10] are used to reduce sunlight reflection. There was also a study that consisted of two or more layers with different roles: a copying plate and a standby window. Additionally various inorganic materials including oxides such as nanoscale BaSO_4_ [11], Cr_2_O_3_ [12], and SiO_2_ [13] are used, as are biomaterials [14] are used as fillers in PRC films. Effective PRC was implemented by increasing the solar reflectance by using the structural properties rather than the material properties of the polymer matrix in a hierarchical structure, such as P(VdF-HFP) [15], TPU [16], and polyurethane [17]. In the case of inorganic materials, these materials are advantageous for radiating heat energy to the outside, but an adhesive layer must be attached to materials that requires cooling, which adversely affects the cooling performance [18,19]. Therefore, in previous studies, organic and inorganic particles were added to a polymer matrix to produce a composite, and the cooling performance of this coating was confirmed. Effective PRC has also been achieved by preparing a nanofiber layer using electrospinning to increase solar reflectance [20,21]. To achieve this, yttrium oxide (Y_2_O_3_) has been used. Y_2_O_3_ has a different refractive index from the matrix and an emittance close to that of silica in the air window area, and is chemically stable. Additionally, effective solar light reflection can be achieved by employing hollow particles to manufacture the structure of the material, increasing the number and area of interfaces where reflection occurs [22,23]. In these previous studies, polydimethylsiloxane (PDMS) was used as the matrix of the PRC film, since its electromagnetic wave radiation due to the coupling vibration of the material occurs in the atmospheric window section. PDMS is a polymer made of inorganic materials, and the Si–O–Si bond is used in radiation cooling research to emit electromagnetic waves in the atmospheric window section. Among the fillers that have an excellent radiant cooling effect, silica has a Si–O–Si bond, like PDMS, making it have a high atmospheric window emissivity and solar reflectance, both of which are optical properties. However, when silica and PDMS are mixed, their refractive indices become similar because they contain similar element contents, but the relative refractive index is low, which is expected to negatively affect solar reflectance. Thus, Y_2_O_3_, which has good atmospheric window emissivity and high solar reflectance, is used instead [24].

In this study, Y_2_O_3_ was synthesized to replace previously used paint fillers, such as silica and barium sulfate (BaSO_4_), for preparing hollow particles. Since Y_2_O_3_ exhibits excellent chemical stability and heat resistance at high temperatures, the exterior coating is expected to maintain high radiative cooling efficiency for an extended period, making it a durable choice. While the Y_2_O_3_ used in previous studies is nano-sized [25], we designed raspberry-like hollow particle sizes that had an impact on the optical properties [23]. The structure of these particles was confirmed, and their structure was compared with the intermediate particles generated during the manufacturing process. The optical properties of the materials generated throughout the manufacturing process were predicted using finite-difference time-domain (FDTD) simulation and compared with those of the final manufactured particles. Additionally, the actual cooling characteristics and cooling power were confirmed using external experiments.

## 2. Experiments

### 2.1. Materials

The particles used in this study were all synthesized in a laboratory, and commercially available BaSO_4_ (Sigma-Aldrich, St. Louis, MO, USA) was used as a reference sample. To confirm the characteristics of the particles, PDMS (Sylgard 184, DOW corning, Midland, MI, USA) was used as the matrix of the film, and formic acid and NaOH (Samchun, Ansan-si, Republic of Korea) were used as the reagents for preparing the particles. Melamine, the formaldehyde, Y(NO_3_)_3_·6H_2_O, and urea were obtained from Sigma-Aldrich. Deionized (DI) water was used as a solvent because it undergoes an aqueous reaction.

### 2.2. Passive Radiative Cooling Film Fabrication

**Figure 1:**
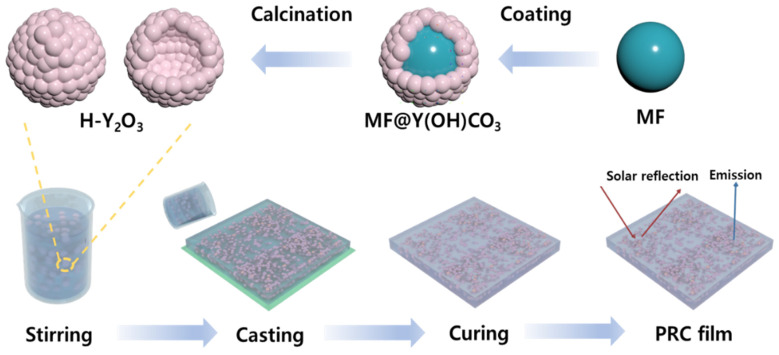
Schematic diagram of the Y_2_O_3_ hollow particle fabrication process used for passive radiation cooling and the PRC film manufacturing process using hollow particles.

To prepare hollow Y_2_O_3_ (H-Y_2_O_3_), a yttrium-coated core–shell structure was first synthesized. The sacrificial template inside was then removed to produce raspberry-like H-Y_2_O_3_. Figure 1 shows the fabrication scheme [26]. An MF (melamine formaldhyde) core was used as the core in the core–shell structure, and a sacrificial template was synthesized. First, formaldehyde, NaOH, and melamine were added to DI water, and the mixture was stirred at 50 °C for 40 min to prepare an MF prepolymer solution. The color of the solution changed from white to transparent. Thereafter, formic acid was added to the condensate to obtain MF cores. At this time, the size of the MF core could be controlled by changing the experimental conditions. The resulting MF was filtered using DI water and EtOH to eliminate small-sized MF. The solvent was then removed using vacuum drying at 60 °C overnight. Subsequently, the MF was sealed and stored at room temperature. A core–shell was fabricated using the manufactured MF. To fabricate the core–shell structure, Y(NO_3_)_3_·6H_2_O, the raw material for Y, MF as a core, and urea were added to DI water, and then the MF was dispersed using ultrasonic treatment. The well-dispersed solution was reacted in a closed state at 85 °C for 3 h to produce a core–shell structure, MF@Y(OH)CO_3_. Thereafter, it was filtered to remove unreacted MF and nano-sized MF@Y(OH)CO_3_ and vacuum-dried to obtain the particles. The core–shell structure was stored under the same conditions as the MF core. Finally, for H-Y_2_O_3_ production, the prepared MF@Y(OH)CO_3_ core–shell structure was heated in a muffle furnace up to 800 °C at a rate of 2 °C/min to remove the sacrificial template; the temperature holding time was 2 h. At this time, oxygen was applied to the furnace, and the sacrificial template MF burned, allowing the shell structure to oxidize to Y_2_O_3_. To prepare the PRC films, 30 vol% of each particle (MF, MF@Y(OH)CO_3_, and H-Y_2_O_3_) generated during the H-Y_2_O_3_ manufacturing process and a filler serving as a reference sample were uniformly mixed with PDMS and stirred. The resulting solution was coated on a glass Petri dish with a thickness of 2 mm and cured in a vacuum at 60 °C to prepare the PRC films.

### 2.3. Optical Property Simulation

FDTD simulations were conducted on the optical properties of the fabricated particles using the Lumerical FDTD simulation program from Ansys (Canonsburg, PA, USA). These simulations are employed to predict the optical properties of the particles. Based on these predictions, the scattering efficiency of the PRC particles in the solar wavelength range, which is a crucial factor for radiative cooling, can be obtained and used as a reference when synthesizing real PRC particles. The electric field distribution obtained using FDTD simulations illustrates the wave energy released via reflection and scattering when a specific wavelength interacts with a PRC particle. Scattering efficiency measures the degree of wavelength scattering as it passes through the particle, and when graphed, higher waviness indicates greater scattering. Additionally, the electric field distribution represents the strength of the electric field generated when a radio wave of a certain wavelength is radiated onto the particle. The lower the electric field strength after passing through the particle, the more effective the wavelength scattering. This simulation can be used to predict the reflectivity of a particle’s sunlight area.

### 2.4. Characteriazation

In this study, an analysis was performed to confirm the characteristics of the particles generated during the H-Y_2_O_3_ manufacturing process. To determine the size distribution of the particles, DLS analysis was performed to determine the changes in the particle size during the manufacturing process. Next, SEM, EDS, and XRD analyses were performed to confirm the morphology and composition of the particles. Additionally, UV–Vis–NIR and FT-IR analyses were performed to confirm the optical properties, which are crucial for radiative cooling. For the UV–Vis–NIR analysis, the UV–Vis–NIR measurement range of 250–2500 nm served as the range in which the sun enters. FT-IR analysis is used to measure the emissivity of a sample in the IR region. PRC film radiates heat energy to the outside in the form of electromagnetic waves. For heat energy to be radiated effectively, electromagnetic waves must pass through the atmosphere. If the wavelength band of the electromagnetic wave is in the absorption part of nitrogen and oxygen, which are the main components of the atmosphere, it does not reach outer space and is absorbed into the atmosphere. Since this goes against the goal of preventing global warming, the wavelength band that is not absorbed is called the atmospheric window and ranges from 8 to 13 μm.

### 2.5. Field Tests

**Figure 2:**
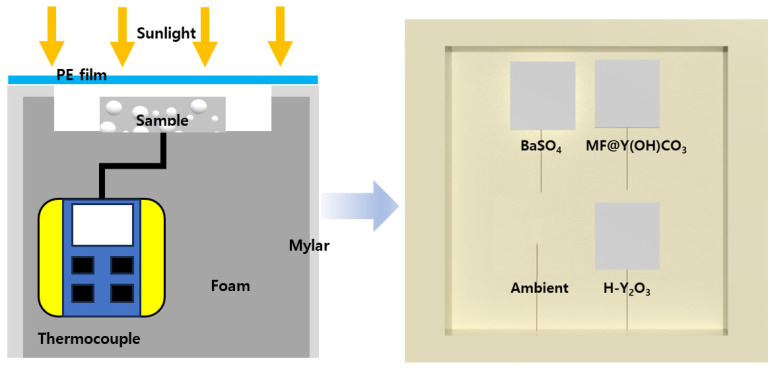
Schematic illustration of the cooling capacity measurement. (**Left**) side, (**right**) top view.

An external experiment was conducted to confirm the cooling power of the PRC film in a real-world environment, and a schematic illustration is provided in Figure 2. The measurement took place at Chung-Ang University in Anseong, Repubilc of Korea, (37°00′27.1′′ N 127°13′48.1′′ E) on 21 July 2023. The field test scheme is depicted in Figure 2. To minimize external light except sunlight, the sides of the foam box were covered with Mylar, and the sample was positioned on top of the box to efficiently reflect and emit sunlight. To measure the temperature change in the samples, a thermocouple was contacted to the bottom of each sample. It was fixed using insulating tape to prevent it from falling, and the measured temperature was collected using a data logger (PCE-T 1200, PCE Instruments, Southampton, UK). Also, to check the ambient temperature, a thermocouple was placed vertically and measured. Additionally, an anemometer and a hygrometer were installed to monitor additional external conditions during the experiment. Before conducting the external experiments, the samples were covered with high-transmittance PE films in the solar and atmospheric window areas to minimize the effects of conduction and convection and control external conditions that could significantly impact the cooling performance.

### 2.6. Cooling Power Calculation

Cooling power is a quantification of how much the PRC film is cooled, and it is the sum of the amount of thermal energy entering and leaving the sample. According to the PRC calculation formula, energy is transferred based on the optical properties of the sample. A material that ideally emits 100% of all wavelengths is called a blackbody, and the radiance of this blackbody ($I_{BB}$) is expressed using Planck’s equation:

(1) $$I_{BB}=({2hc}^{2}/\lambda^{5})/\;[e^{hc/\left(\lambda \kappa_{B}T\right)}-1],$$

where h is Planck’s constant, c is the speed of light, λ is the length of the wavelength emitted by the blackbody, $\kappa_{B}$ is the Boltzmann constant, and *T* is the temperature of the sample. The radioactivity of the sample can be calculated using $I_{BB}$ as follows:

(2) $$P_{rad}\left(T\right)=A\int d\Omega cos\theta \int_{0}^{\infty }d\lambda I_{BB}\left(T,\lambda \right)\varepsilon \left(\lambda ,\theta \right),$$

where *A* is the area of the sample, and ε(λ, θ) is the emissivity of the PRC film expressed as $\varepsilon_{atm}\left(\lambda ,\theta \right)={1-t(\lambda )}^{1/cos\theta }$. Here, t(λ) is the transmittance of the atmospheric window area calculated using the ATRAN modeling software (Lord, S.D. 1992, NASA Technical Memor. 103957, G173-03 Reference Spectra Derived from SMARTS v. 2.9.2). Elements that hinder cooling enter via conduction or convection, which are energy transfer methods, not radiation. The energy coming from outside due to conduction and convection is expressed as

(3) $$P_{non,rad}=Ah_{c}\left(T_{amb}-T_{sam}\right)$$

$h_{c}$ is a thermal coefficient and changes according to the external temperature, humidity, and wind speed. To calculate this, the actual measured sample temperature can be substituted into the cooling power formula to calculate the instantaneous thermal coefficient. The energy entering the sample via radiation is $P_{atm}(T_{amb})$ around the sample and $P_{Sun}$ from the sun. The radiant energy from the surroundings is assumed to come from the air, and solar energy enters the sample through the atmosphere at 250 nm to 2.5 μm in the UV-visible-near-infrared range. This is expressed as:

(4) $$P_{atm}(T_{amb})=A\int d\Omega cos\theta \int_{0}^{\infty }d\lambda I_{BB}(T_{amb},\lambda )\varepsilon (\lambda ,\theta )\varepsilon_{atm}(\lambda ,\theta )$$

(5) $$P_{Sun}=A\int_{0}^{\infty }d\lambda \varepsilon (\lambda ,\theta )I_{AM1.5}(\lambda )$$

The expression $I_{AM1.5}$ in $P_{Sun}$ is the absorbance received from the sun and is calculated in the same way as (1). The passive radiant cooling power $P_{net}$, which is the sum of the above equations, is summarized in Equation (6).

(6) $$P_{net}(T)=P_{rad}(T)-P_{Sun}-P_{non,rad}-P_{atm}(T_{ambient})$$

To increase the cooling power, the energy input from the outside must be reduced and the energy emitted must be increased.

## 3. Results and Discussion

### 3.1. Morphology of the Hollow Particles

**Figure 3:**
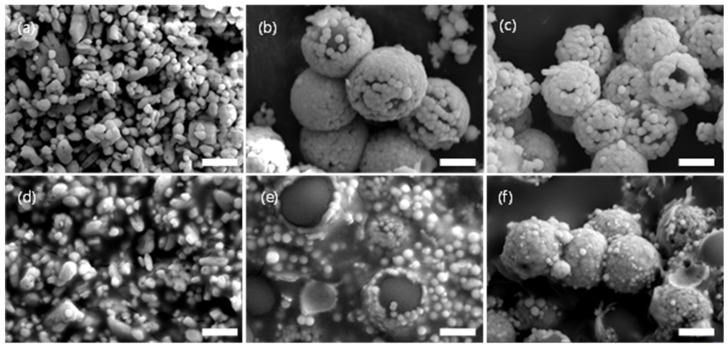
(**a**–**c**) SEM images of the PRC particles and (**d**–**f**) cross-sectional SEM images of the PRC films: (**a**,**d**) BaSO_4_, (**b**,**e**) MF@Y(OH)CO_3_, and (**c**,**f**) H-Y_2_O_3_. The scale bar is 1 μm.

**Figure 4:**
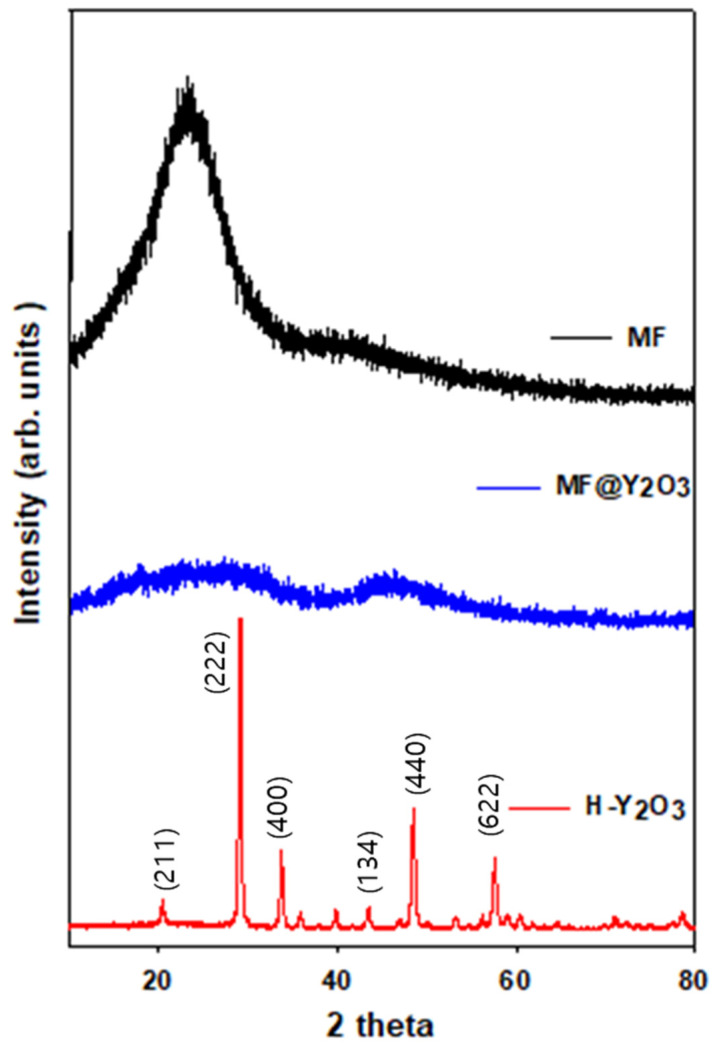
XRD spectra of the particles generated during the fabrication of H-Y_2_O_3_.

As shown in Figure 3, SEM analysis was performed to confirm the characteristics of the PRC filler particles. Figure 3a shows the image of BaSO_4_ as a reference material, and Figure 3d shows the SEM cross-sectional image of the film. BaSO_4_ had particle sizes of 1 μm. Figure 3b shows the SEM image of MF@Y(OH)CO_3_ with the core–shell structure. The size of the MF particles constituting the core was 2.5 μm, and the particle size of Y(OH)CO_3_ constituting the shell was 200 nm, which is at the nano level. Figure 3e shows the image of the MF@Y(OH)CO_3_ particles used in the PRC film, and it was confirmed that the core–shell structure was maintained during the manufacturing process. Figure 3c,f depict images of the H-Y_2_O_3_ hollow particles. During the calcination of H-Y_2_O_3_, the MF was removed, and Y(OH)CO_3_ was transformed into Y_2_O_3_ in the shell structure. It was confirmed that the particle size was reduced to 2.3 μm when compared to that of the MF@Y(OH)CO_3_ size at 2.7 μm. XRD analyses were conducted to confirm the morphology and composition of the particles used in the PRC film. Broad peaks were observed in the XRD spectra of MF and MF@Y(OH)CO_3_ (Figure 4). However, calcined H-Y_2_O_3_ exhibited intensity at peaks (211), (222), (400), (440), (134), (440), and (622) of Y_2_O_3_, confirming that only Y_2_O_3_ remained.

Figures S1–S3 present the EDS images of the particles. Figure S1 depicts the presence of C, N, and O elements in the MF, while Figure S3 depicts the presence of N in the MF, which divides the core of MF@Y(OH)CO_3_ (Figure S2c) and the shell (Figure S2d). The presence of the Y element was verified to confirm whether the core–shell particles were well synthesized. Figure S4 shows the EDS images of Y_2_O_3_. C and N are absent in Figure S3b, while Y and O are present in Figure S3c,d, confirming that MF was removed by calcination. Using this morphology confirmation, it was confirmed that MF@Y(OH)CO_3_ has a core–shell structure and that calcinated H-Y_2_O_3_ has a hollow structure.

### 3.2. Optical Property Simulation

**Figure 5:**
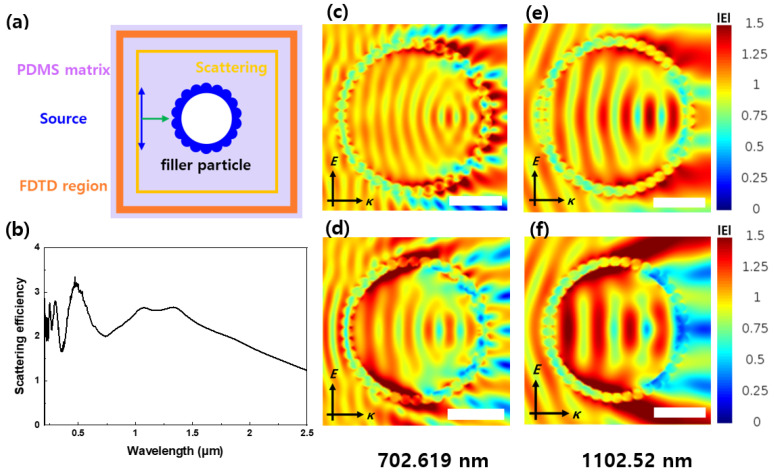
(**a**) Schematic of the FDTD simulations. (**b**) Scattering efficiency of the H-Y_2_O_3_ particles. Electric field distributions of (**c**,**e**) MF@Y(OH)CO_3_ and (**d**,**f**) H-Y_2_O_3_. The scale bar is 1 μm.

FDTD simulations were conducted to confirm the optical properties of the PRC particles. In Figure 5a, the FDTD simulation settings for the particles are depicted. We used PDMS as the matrix for the PRC films, established the FDTD simulation and scattering intervals, and placed the PRC filler particles in the center.

Figure 5b displays the particle scattering efficiency for each wavelength in the FDTD cross-section. The scattering efficiency wavelength range was set to 0–2500 nm. H-Y_2_O_3_ exhibited a higher total scattering efficiency in 0–2500 nm, indicating its expected higher solar reflectance compared to Y(OH)CO_3_.

Figure 5c–f show the electric field distribution. The intensity of the electric field corresponds to the wavelength intensity, allowing judgment of the electric field distribution. The wavelength source enters from the right side and moves to the left in the image. In Figure 5c,d, the electric field distribution of Y(OH)CO_3_ and H-Y_2_O_3_ at a wavelength of 702.619 nm is shown. The Y(OH)CO_3_ electric field magnitude increases after passing through the particle due to interference. However, in the case of H-Y_2_O_3_, the electric field on the left is larger, and the electric field on the right is smaller than that of Y(OH)CO_3_. When the wavelength is transmitted to the particle, H-Y_2_O_3_ reflects a relatively larger amount than Y(OH)CO_3_, leading to the expected solar reflection. Figure 5e,f show similar trends for a wavelength length of 1102.52 nm. Therefore, H-Y_2_O_3_ is expected to have a higher solar reflectance than Y(OH)CO_3_.

### 3.3. Properties of Passive Radiative Cooling Films

**Figure 6:**
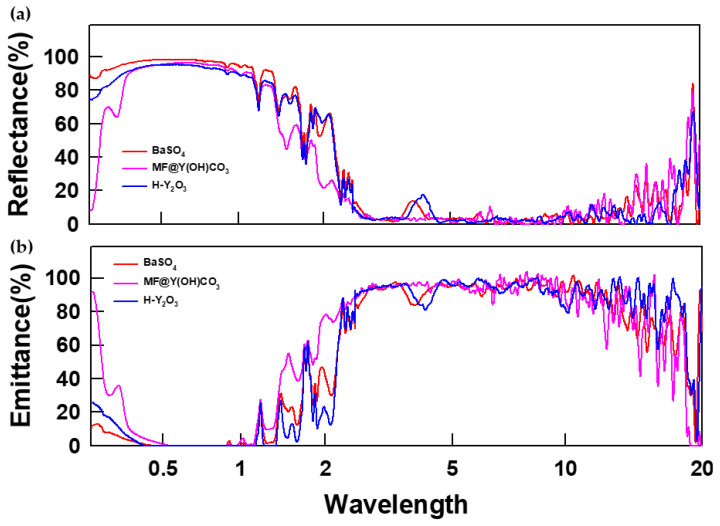
Optical properties of the PRC films: (**a**) reflectance and (**b**) emittance.

Figure 6 and Figure S4 depict the optical properties of the PRC films. To demonstrate the flexibility of the PRC film, bent and restored images of the sample are shown in Figure S4a–c, confirming the flexibility of all the PRC films, including the reference film. In Figure 4a, the reflectance graph indicates that higher solar reflectance in the 250 nm–2.5 μm region, where solar radiation exists, and higher emissivity in the 2.5–20 μm region, where atmospheric window transmittance exists, resulting in higher cooling power. The average reflectance in the sunlight region was evaluated, with BaSO_4_ having the highest value at 74.24%, H-Y_2_O_3_ at 70.73%, and MF@Y(OH)CO_3_ at the lowest value of 60.42%. Compared to the FDTD simulation results, we can see a trend that H-Y_2_O_3_ is higher than MF@Y(OH)CO_3_, which confirms that H-Y_2_O_3_ particles have a direct effect on increasing solar reflectance. Figure 4b shows the analysis of emissivity in the atmospheric window section, with H-Y_2_O_3_ having the highest average value of 86.24%, followed by BaSO_4_ and MF@Y(OH)CO_3_.

### 3.4. Field Tests

**Figure 7:**
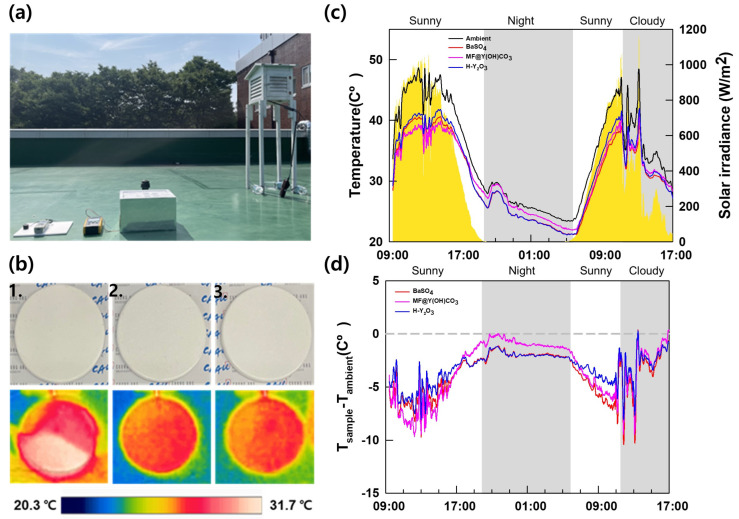
(**a**) Field test setup for the cooling capacity measurement. (**b**) IR camera image captured at 14:00 on 21 July: 1. BaSO_4_, 2. MF@Y(OH)CO_3_, and 3. H-Y_2_O_3_. (**c**) Sample temperature and solar irradiance. (**d**) Differences between the ambient and sample temperatures.

Field tests were conducted to confirm the actual cooling performance. Figure 7a shows the experimental device used for the measurements. In Figure 7b, an IR camera image captured at 14:00 on 21 July 2023 shows the external temperature of the sample. BaSO_4_ exhibited the highest external temperature at 26.8 °C, followed by MF@Y(OH)CO_3_ and H-Y_2_O_3_, which showed lower temperatures. Figure 7c displays the sample temperature and solar irradiance, while Figure 7d presents a graph showing the temperature difference between the ambient temperature and the sample temperature. Lower values indicate cooler sample temperatures. The insolation and temperature trends of the measured PRC films were similar, and no interference other than sunlight was observed. The gray area represents periods of low insolation during nighttime and cloudy weather. During the daytime, the MF@Y(OH)CO_3_ sample exhibited the lowest temperature, followed by BaSO_4_ and H-Y_2_O_3_. This is because the PRC is significantly influenced by solar reflectance during the daytime. The core material of MF@Y(OH)CO_3_, MF, has a thermal conductivity of 0.03 W/m∙K, reducing heat conduction from the surroundings and making it less affected by external temperatures. As a result, the PRC film with MF@Y(OH)CO_3_ was measured to have a lower temperature than H-Y_2_O_3_. The nighttime trend differed from the daytime trend. BaSO_4_ and H-Y_2_O_3_ cooled to −3 °C with a slight difference, while the temperature decrease of MF@Y(OH)CO_3_ was less significant. This showed a similar trend to the atmospheric window emissivity measured using FDTD simulation and FT-IR, and nighttime passive radiative cooling was found to be proportional to the atmospheric window emissivity.

### 3.5. Cooling Power Calculation

**Figure 8:**
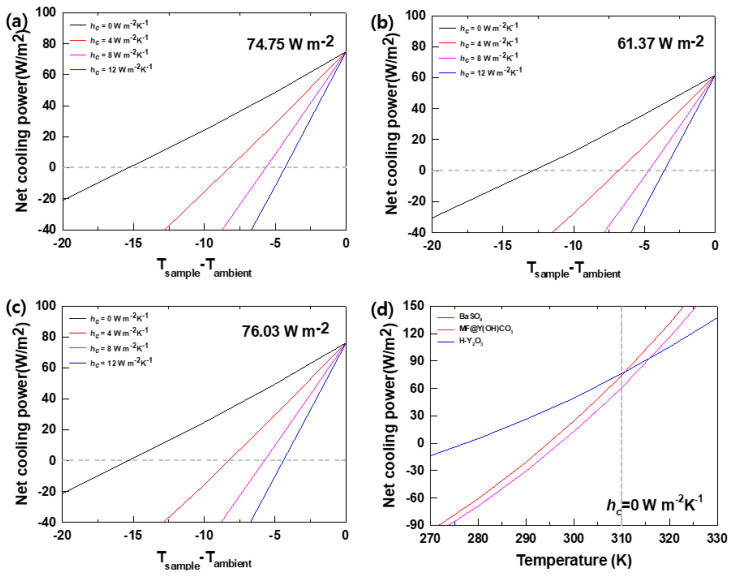
Calculation of the cooling power and theoretical cooling power of the PRC films at an ambient temperature of 310 K. Heat transfer coefficient (*h_c_*) values of 0, 4, 8, and 12 W·m^−2^·K^−1^ were used in the calculations: (**a**) BaSO_4_, (**b**) MF@Y(OH)CO_3_, and (**c**) H-Y_2_O_3_. (**d**) Calculation of the cooling power according to the temperature of the PRC film. Here, an (*h_c_*) value of 0 W·m^−2^·K^−1^ was applied.

The cooling power was calculated based on the optical properties of the PRC film and temperature data obtained from the field tests. Equations (1)–(6) were used for the calculations, along with arbitrary heat transfer coefficient ($h_{c}$) values of 0, 4, 8, and 12 W·m^−2^·K^−1^. Figure 8 images the net cooling power obtained using these calculations. (a) to (c) are shown in the order of BaSO_4_, MF@Y(OH)CO_3_, and H-Y_2_O_3_, respectively, and the maximum cooling power was confirmed to be unrelated to the heat transfer coefficient values ($h_{c}$). And (d) is a graph summarizing the maximum cooling power according to the external temperature of each sample. By specifying the temperature on the graph, you can calculate the maximum cooling power at that time. The ambient temperature, based on the field test measurements, was set to 310 K. H-Y_2_O_3_ exhibited the highest cooling power at 76.03 W·m^−2^, followed by BaSO4 at 74.75 W·m^−2^, and MF@Y(OH)CO_3_ at 61.37 W·m^−2^. The actual measured temperature allowed the confirmation of an $h_{c}$ value of 0 W·m^−2^ · K^−1^ using field testing, demonstrating the effectiveness of the PE film in preventing conduction and convection in the atmosphere. Figure 8d summarizes the cooling powers of the PRC films. BaSO_4_ showed the highest cooling power above 310 K, while H-Y_2_O_3_ exhibited the lowest cooling power below 310 K. MF@Y(OH)CO_3_ displayed a higher cooling power than H-Y_2_O_3_ at temperatures of 315 K or higher. These results confirm that the PRC filler with the highest passive radiant cooling capacity varies depending on the external temperature.

## 4. Conclusions

Passive radiative cooling, a study aimed at reducing energy consumption, is where atmospheric window radiation is mainly achieved using a polymer matrix. In the case of polymers with good atmospheric window radiation, there are limits to radiative cooling during the day because of their low solar reflectance. To improve this, the shape and material of the additive must be tactically selected. To overcome this, the cooling power can be calculated according to the shape of the particles to produce a PRC film optimized according to the temperature conditions. In addition, because it does not consume energy and has the advantage of being applicable externally, research is being conducted to apply it not only to buildings but also to automobiles and clothing. These studies simultaneously seek to improve cooling power.

In this study, FDTD simulations were conducted to confirm whether the optical properties vary depending on the morphology of the fillers used for PRC. Using FDTD simulation can help avoid trial and error and excessive process design during the particle design phase. The optical properties of the actual samples were compared using analyses. We confirmed the material properties of the particles at each stage of the hollow particle manufacturing process and those of the final particles. The optical data revealed that sacrificial MF exhibited low solar reflectance and atmospheric window emissivity due to the molecular bond vibrations of N = C and N–C in the molecule. The sample with the highest solar reflectance was BaSO_4_, measuring 74.24%, and the PRC film with the highest atmospheric window emissivity was H-Y_2_O_3_ (Y–O–Y), a hollow structure with a value of 86.24%. H-Y_2_O_3_ also exhibited a higher solar reflectance than the MF@Y(OH)CO_3_ core–shell structure. The results of the external test showed that MF@Y(OH)CO_3_ had the lowest temperature of 25.4 °C during the day, followed by H-Y_2_O_3_ and BaSO_4_. This is due to the low thermal conductivity of the core MF. At night, H-Y_2_O_3_ recorded the lowest temperature of 22.5 °C, followed by BaSO_4_ and MF@Y(OH)CO_3_. This indicates that H-Y_2_O_3_ has the highest atmospheric window emissivity. Cooling power measurements showed that H-Y_2_O_3_ had a higher cooling power (76.03 W·m^−2^) compared to the reference sample used as a conventional paint filler (74.75 W·m^−2^). Through this, it was confirmed that hollow particles have a superior cooling performance compared to the core cell particles manufactured during the hollow particle synthesis process. FDTD simulations also help prevent the over-designing of the process by analyzing the optical properties of particles generated at each step of the manufacturing process. By calculating the cooling power according to the shape of the particles, a PRC film optimized for temperature conditions can be produced.

## Data Availability

The datasets used and analyzed during the current study are available from the corresponding author on reasonable request.

## Supplementary Materials

The following supporting information can be downloaded at https://www.mdpi.com/article/10.3390/ma16237373/s1: Figure S1: SEM–EDS images of the MF particles: (a) SEM image, (b) C, (c) N, and (d) O; Figure S2: SEM-EDS images of the MF@Y(OH)CO_3_ particles: (a) SEM image, (b) C, (c) N, and (d) Y; Figure S3: SEM-EDS images of the H-Y_2_O_3_ particles: (a) SEM image, (b) C, (c) O, and (d) Y; Figure S4: Optical images of the flexibility of the (a) BaSO_4_, (b) MF@Y(OH)CO_3_, and (c) H-Y_2_O_3_ PRC fillers.
