# Supplementary material for: Fabrication of Yttrium Oxide Hollow Films for Efficient Passive Radiative Cooling
Source: Materials (Basel). 2023 Nov 27;16(23):7373. doi: 10.3390/ma16237373 (PMC10707271; doi:10.3390/ma16237373)
Supplement: Supplementary file 1 [file materials-16-07373-s001.zip › materials-2692390-supplementary.pdf]

Supplementary

Fabrication of Yttrium Oxide Hollow Films for Efficient Passive Radiative Cooling

Heegyeom Jeon [1], Sohyeon Sung [2], Jeehoon Yu [1], Hyun Kim [2], Yong Seok Kim [2] and Youngjae Yoo [1,*]

[1] Department of Advanced Materials Engineering, Chung-Ang University, Anseong 17546, Republic of Korea; avel004@cau.ac.kr (H.J.); yujeehoon@cau.ac.kr (J.Y.)
[2] Advanced Materials Division, Korea Research Institute of Chemical Technology (KRICT), Daejeon 34114, Republic of Korea; gus6319@krict.re.kr (S.S.); hyunkim@krict.re.kr (H.K.); yongskim@krict.re.kr (Y.S.K.)
* Correspondence: yjyoo@cau.ac.kr

3 pages in total, including 4 figures.

Table of contents

Figure S1 SEM–EDS images of the MF particles: (a)SEM image, (b) C, (c) N, and (d) O

Figure S2 SEM-EDS images of the MF@Y(OH)CO_3 particles: (a) SEM image, (b) C, (c) N, and (d) Y

Figure S3 SEM-EDS images of the H-Y_2O_3 particles: (a) SEM image, (b) C, (c) O, and (d) Y

Figure S4. Optical images of the flexibility of the (a) $BaSO_4$, (b) MF@Y(OH)CO_3, and (c) H-Y_2O_3 PRC fillers

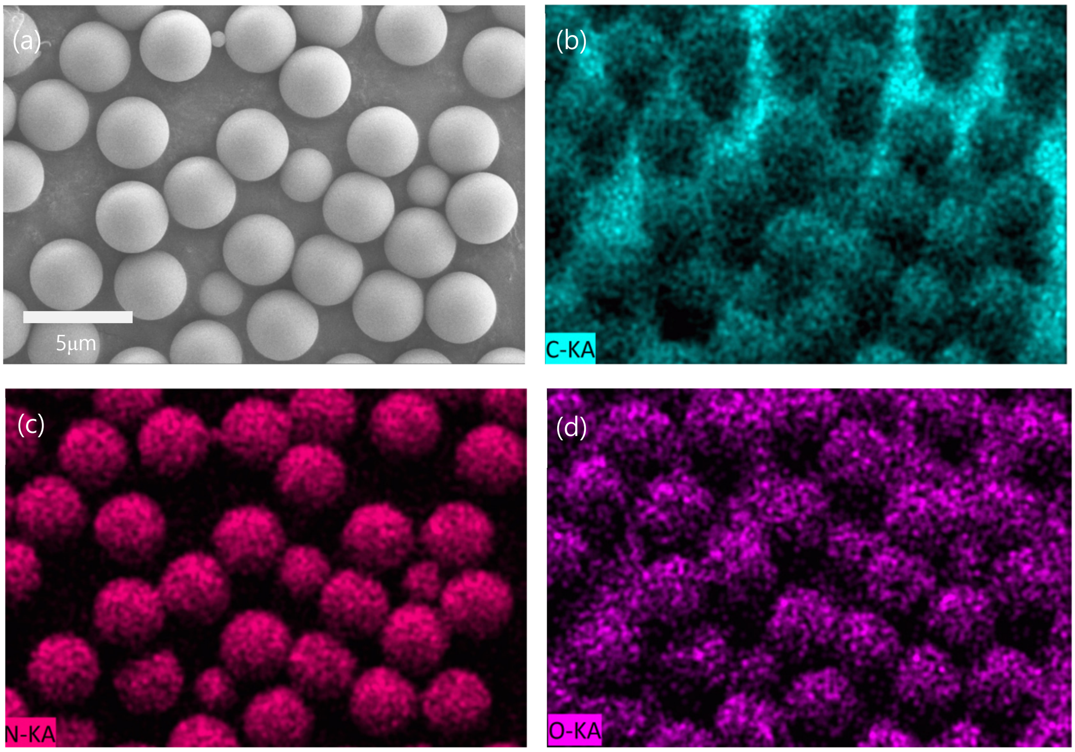

Figure S1 SEM-EDS images of the MF particles: (a)SEM image, (b) C, (c) N, and (d) O

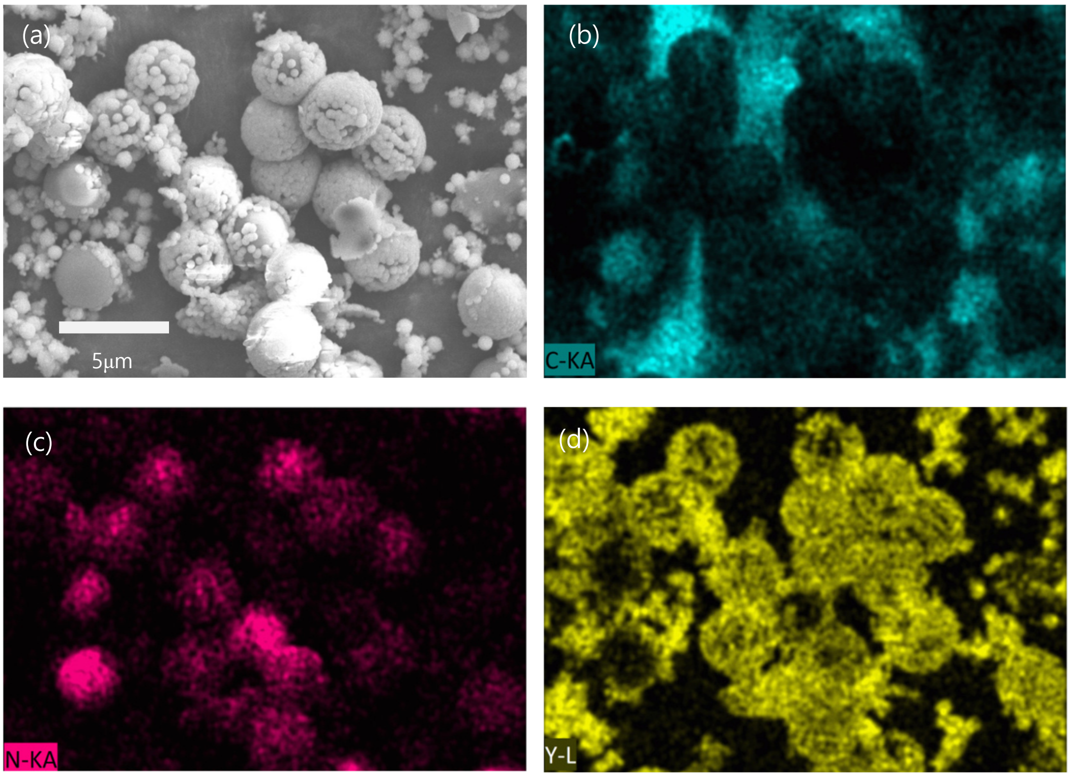

Figure S2 SEM-EDS images of the MF@Y(OH)CO_3 particles: (a) SEM image, (b) C, (c) N, and (d) Y

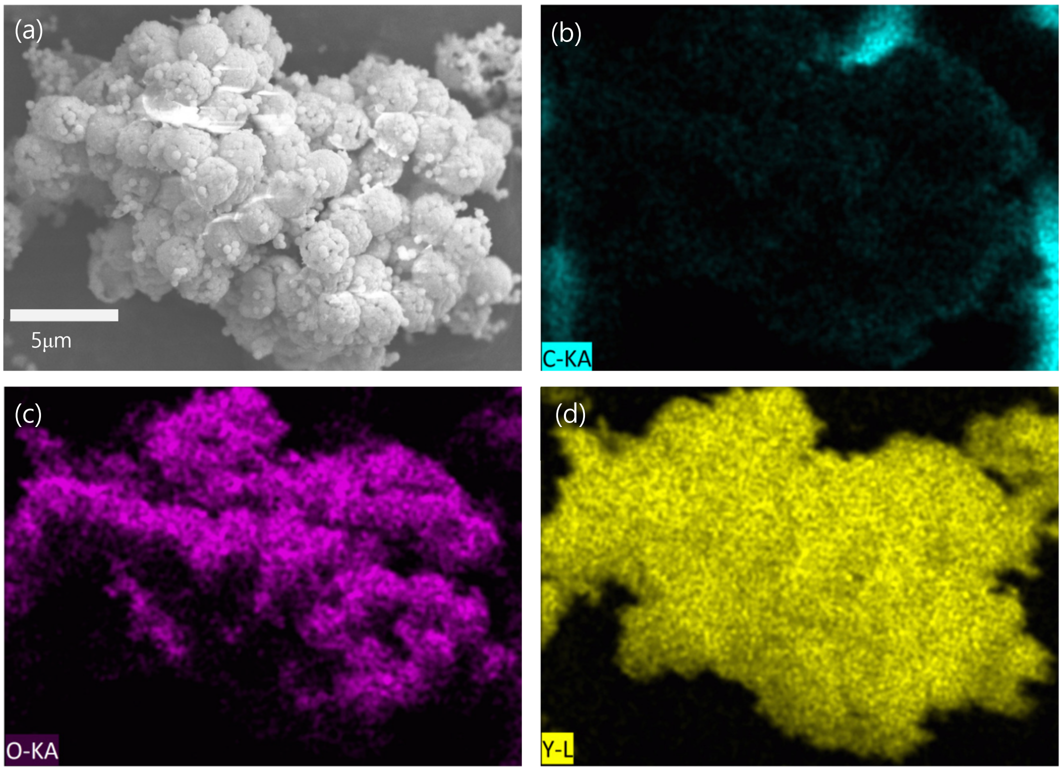

Figure S3 SEM-EDS images of the H-Y_2O_3 particles: (a) SEM image, (b) C, (c) O, and (d) Y

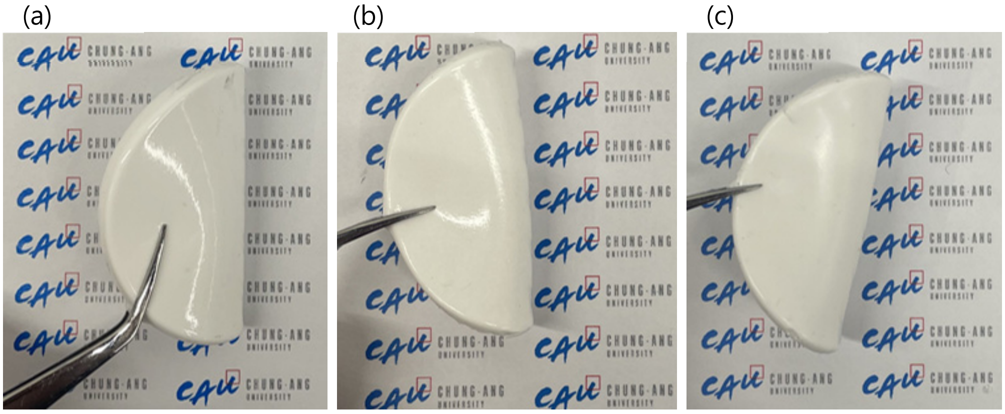

Figure S4. Optical image of (a)$BaSO_4$, (b) MF@$(OH)CO_3$, (c) H-Y_2O_3,
